# Effects of a 4‐Week Multi‐Exercise Isometric Resistance Training Programme on Resting and Ambulatory Blood Pressure in Normotensive Adults

**DOI:** 10.1002/ejsc.70202

**Published:** 2026-06-06

**Authors:** Ben H. Wright, Mark R. Antrobus, Peter G. W. Jones, Anthony W. Baross

**Affiliations:** ^1^ Department of Sport, Health Sciences and Social Work Oxford Brookes University Oxford UK; ^2^ School of Education, Childhood, Youth & Sport, Faculty of Wellbeing, Education and Language Studies The Open University Milton Keynes UK; ^3^ Glasgow Caledonian University London UK; ^4^ Centre for Physical Activity and Life Sciences, Faculty of Art, Science and Technology University of Northampton Northamptonshire UK

**Keywords:** ambulatory blood pressure, blood pressure, isometric exercise, isometric handgrip, isometric training band

## Abstract

Isometric resistance training (IRT) can reduce resting and ambulatory blood pressure (AmBP) yet established exercise methods lack versatility and may present with participation barriers. An isometric training band (ITB) has been identified as an alternative modality; however, its long‐term effects on resting and ambulatory blood pressure (BP) remains unknown. This study assessed the effects of the ITB on resting BP and AmBP following 4 weeks of IRT. Forty‐two normotensive adults (22 male, 20 female; mean ± SD, age 31 ± 14 years, systolic [sBP], 120 ± 5 mmHg, diastolic [dBP], 72 ± 7 mmHg), were randomised to a control (CON), isometric handgrip (IHG) or ITB group. Resting (systolic, diastolic, mean arterial BP and heart rate [HR]) and AmBP (24‐h, daytime and night‐time) were measured pre‐ and post‐4 weeks of supervised IRT (4 x 2‐min contractions at 30% MVC [IHG] or 4 x 2‐min contractions at CR‐10 values equivalent to 30% MVC [ITB]). Resting sBP was reduced for IHG (−4.6 mmHg, *p* < 0.05) and ITB (−4.5 mmHg, *p* < 0.05) following 4 weeks of IRT, alongside significant within‐group differences in night‐time sBP for both training groups (ITB −4 mmHg, IHG, −4.7 mmHg). No differences were seen within or between groups for resting dBP, mBP, HR and 24‐h and daytime AmBP (*p* > 0.05 across all measures). These findings suggest 4 weeks of IRT using the ITB can elicit reductions in resting BP and night‐time AmBP in normotensives comparable to IHG, indicating the ITB may offer a practical and cost‐effective alternative to established interventions.

## Highlights

- Isometric resistance training can reduce resting and ambulatory blood pressure, yet traditional methods require the use of specialised equipment, lack exercise versatility or require adopting exercise positions that may be challenging, limiting accessibility.
- Our results suggest that the use of an inexpensive novel isometric training band can elicit significant reductions in resting and night‐time systolic blood pressure following a 4‐week intervention.
- Blood pressure reductions elicited from the isometric training band were comparable to validated isometric handgrip interventions, therefore use of the ITB protocol may provide an accessible, versatile and cost‐effective alternative to conventional isometric resistance training approaches.

## 1 Introduction

High blood pressure (BP) is a significant global condition estimated to be prevalent in 1.3 billion people (Zhou et al. 2021) attributable to a fifth of global deaths (∼10 million) and 235 million disability‐adjusted life years annually (Abbafati et al. 2020). Left uncontrolled elevated systolic blood pressure (sBP) is the leading risk factor for cardiovascular disease‐related mortality (Abbafati et al. 2020) and a significant risk factor for cerebrovascular disease (Webb and Werring 2022) ischaemic stroke, intracerebral haemorrhage and chronic kidney disease (Burnier and Damianaki 2023). Despite effective lifestyle interventions (Carey et al. 2021), including physical activity and pharmacotherapy, adherence and compliance remain low with global estimates of only ∼17% of adults meeting physical activity guidelines (Garcia‐Hermoso et al. 2023) and ∼50% discontinuing initial pharmacological treatment within a year of prescription (Vrijens et al. 2008). Therefore, alternative efficacious and adherable strategies are required to combat this global public health challenge.

Isometric resistance training (IRT) is an established nonpharmacological therapeutic intervention to manage hypertension (Edwards et al. 2024), with significant BP reductions achieved with reduced time requirements (∼24‐min per week) compared to commonly prescribed exercise guidelines (150‐min per week). Given a ‘lack of time’ is a significant barrier to physical activity participation (Healy et al. 2024), IRT may offer a practical alternative. Despite evidence supporting reductions in resting BP, the impact on ambulatory blood pressure (AmBP) is largely confined to prehypertensive and hypertensive populations (Pagonas et al. 2017; Stiller‐Moldovan et al. 2012; Taylor et al. 2019) with only Baross et al. (2024) and Somani et al. (2017) reporting significant reductions in young normotensive cohorts. Yet, with the prevalence of hypertension rising amongst younger populations (Haseler et al. 2023) and the heightened lifetime risk associated with elevated BP at a younger age (Hamrahian and Falkner 2022), AmBP represents an important clinical outcome for investigation. In normotensives, AmBP may provide greater clinical value for establishing risk and assessing the preventive effects of exercise interventions as it can establish hypertension phenotypes that remain undetected during resting office measurements, such as white‐coat and masked hypertension (Viera and Shimbo 2014). Furthermore, AmBP enables the assessment of additional parameters, including ambulatory arterial stiffness index (AASI) and blood pressure variability (BPV). The AASI provides an indirect assessment of vascular stiffness (Li et al. 2006), thus allows insight into potential vascular adaptation. Additionally, BPV can be calculated from individual 24‐h ambulatory profiles and is a clinically relevant marker associated with hypertension mediated organ damage, mortality and future development of hypertension (Parati et al. 2020; Sega et al. 2002; Stevens et al. 2016). Collectively, these measures may help to elucidate the mechanisms through which isometric resistance training induces vascular adaptations and reduces long‐term hypertension risk.

Amongst IRT modalities, isometric handgrip (IHG) interventions are the most widely cited, evidencing significant reductions in resting BP irrespective of hypertensive status (Edwards et al. 2023). Alternate methods (wall squat and bilateral leg extension) have demonstrative BP lowering capabilities (Baross et al. 2013; Wiles et al. 2017) though may present with participation barriers as bilateral leg extension requires the use of specialised inaccessible equipment occurring a high cost, and necessitates trained personnel for use. Whereas the isometric wall squat has been described as ‘difficult’ and ‘challenging’ (Roberts et al. 2024), and require individuals to perform an incremental exercise test for intensity prescription which has been considered “complicated’’ and likely inaccessible for individuals with mobility limitations (Roberts et al. 2024). Moreover, all current established IRT methods have reduced exercise variety, which may hinder long‐term adherence (Bachmann et al. 2018). Thus, a recognised and inexpensive isometric training band (ITB) may enhance accessibility to IRT and offer a portable and practical alternative (Wright et al. 2025). The ITB has the capacity to facilitate a range of isometric exercises of differing and multiple muscle groups, with the versatility of exercise choice having implications for long‐term exercise adherence (Wulf et al. 2014). Although recently validated against IHG (Wright et al. 2025), it remains unknown if an ITB training intervention will elicit a reduction in resting or AmBP. Moreover, it is of clinical interest to examine if a multiexercise ITB protocol utilising larger muscle groups may elicit superior BP reductions compared to conventional single exercise IRT modalities (Seals et al. 1983). Hence, it is important to ascertain the efficacy of the ITB protocol to reduce resting and AmBP and to compare to volume‐matched established IRT modalities.

Therefore, the aims of this study were to determine if resting BP and AmBP are reduced following a 4‐week ITB intervention and to examine if effects on BP are comparable to a volume‐matched validated IHG protocol. It was hypothesised that the ITB protocol would elicit significant BP and AmBP reductions when compared to a volume‐matched IHG protocol following a 4‐week intervention.

## 2 Materials and Methods

### 2.1 Participants

**FIGURE 1:**
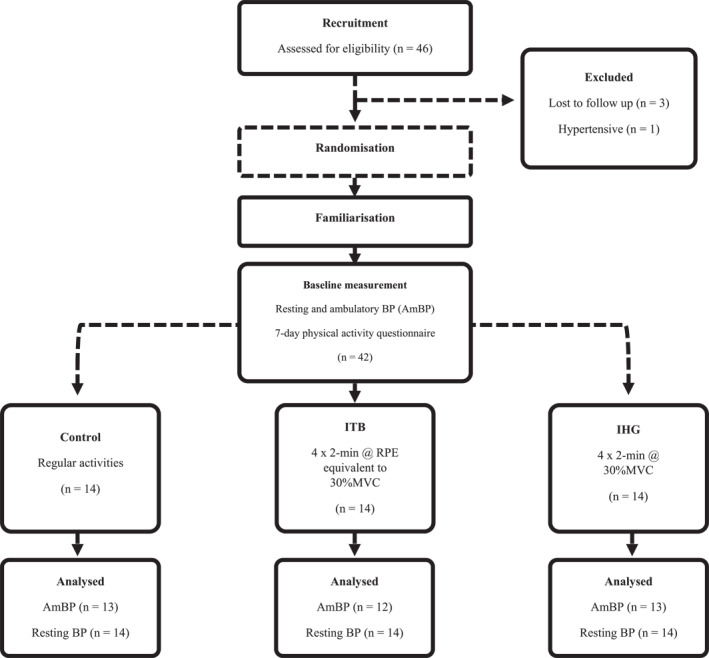
Schematic diagram of the study protocol including participant exclusion, randomisation, allocation of group and completed measurement. AmBP, ambulatory blood pressure measurement; BP, blood pressure; IHG, isometric handgrip; ITB, isometric training band; MVC, maximal voluntary contraction; RPE, rating of perceived exertion.

Forty‐two healthy normotensive adults (22 male, 20 female, [mean ± SD], age 31 ± 14 years, height 170 ± 10 cm, mass 75 ± 16 kg, systolic [sBP], 120 ± 5 mmHg, diastolic [dBP], 72 ± 7 mmHg, mean arterial [mBP], 88 ± 6 mmHg, heart rate [HR], 67 ± 12 bpm^·−1^) were recruited and randomised to a control (CON), IHG or ITB group (Figure 1) using block randomisation through an online number randomiser (Randomizer.org). As evidence suggests that sex does not influence the magnitude of BP reductions following IRT (Bentley et al. 2015; Somani et al. 2017), randomisation was not stratified by sex. Prior to the initial data collection session, participants provided written informed consent and completed a physical activity readiness questionnaire to determine eligibility. Participants were excluded if classified as pre or hypertensive (≥ 130 mmHg sBP and/or ≥ 85 mmHg dBP) from the initial baseline measurement of resting BP, if presenting with a diagnosed cardiovascular, metabolic or respiratory disease (type 1 or type 2 diabetes, CVD, stroke, atrial fibrillation and chronic obstructive pulmonary disease) or a musculoskeletal impairment preventing the ability to complete isometric exercise.

### 2.2 Study Design

The study employed a prospective randomised control trial design. Participants were nonsmokers, nonmedicated and recreationally active by self‐reported physical activity levels (IPAQ‐SF, Table 1) and were instructed to maintain current habitual physical activity levels throughout the duration of the study. Adherence to habitual physical activity was confirmed through verbal confirmation, alongside the comparison of physical activity levels pre and postintervention. Participants were initially familiarised on each measurement procedures, alongside interpretation of the category‐ratio Scale (CR‐10), in addition to the assigned exercise modality if randomised to an exercise group. The CR‐10 scale was selected given its development to be used for localised muscle exertion, and in its application in eliciting comparable responses to the widely prescribed IHG protocol (Wright et al. 2025). Recruitment was completed through advertisement on social media, word of mouth and organisational internal communications. Baseline measurements were completed a minimum of 24‐h from participants' previous physical activity session and for female participants during the same phase of the menstrual cycle. With postmeasurements completed ≥ 24‐h from the final isometric training session to prevent potential influence of exercise‐induced hypotensive effects (Chen and Bonham 2010). Before each visit, participants verbally verified adherence to pretesting requirements, which required avoiding strenuous exercise for the preceding 24 h, refraining from alcohol and caffeine for 12 h and maintaining a 4‐h fasting period (Kallioinen et al. 2017). Institutional ethical approval was obtained (ETH1920‐ 0152) in accordance with the latest Declaration of Helsinki. To estimate sample size, a priori power analyses were completed via G*Power (v.3.1 Düsseldorf, Germany), with effect size measures (Cohen's *d*) calculated (1.2) from previous isometric resistance training studies of comparable duration (4 weeks) sampling normotensive adults (Baddeley‐White et al. 2019; Devereux et al. 2010) a total sample size of 34 participants was required. Accounting for an anticipated attrition rate of 20% (Linke et al. 2011), the final estimated sample size was set at 42 participants.

**TABLE 1:** Comparison of participant baseline characteristics.

Measure	Control (*n* = 14)	ITB (*n* = 14)	IHG (*n* = 14)
Age (years)	36 ± 15	28 ± 13	29 ± 12
Mass (kg)	73 ± 17	79 ± 20	74 ± 13
Resting sBP (mmHg)	119 ± 6.6	122 ± 5	121 ± 6
Resting dBP (mmHg)	73 ± 8.3	71 ± 7.2	74 ± 4.3
Resting mBP (mmHg)	89 ± 7.3	88 ± 5	90 ± 6
Resting HR (bpm^·−1^)	68 ± 11	66 ± 12	68 ± 14
PA^Mod^ (MET‐min/week)	557 ± 1162	754 ± 1546	642 ± 816

*Note:* Data are presented as mean ± standard deviation.

Abbreviations: dBP, diastolic blood pressure; HR, heart rate; IHG, isometric handgrip; ITB, isometric training band; mBP, mean arterial pressure; PA^Mod^, moderate domain physical activity; sBP, systolic blood pressure.

### 2.3 Procedures

#### 2.3.1 Resting Blood Pressure Measurement

Resting BP (sBP, dBP and mBP) was measured using a digital automatic oscillometric BP device (Omron HEM‐907, Omron Healthcare, Japan). Resting BP was measured by placing the BP cuff covering 80% of the upper left arm ∼1.5 cm above the antecubital fossa and with the artery position marker set in line with the brachial artery. During measurement, participants were seated with legs uncrossed, feet flat and with their back supported, the upper arm was rested and supported at mid‐sternal level. After 10‐min of uninterrupted seated rest, three blood pressure readings were taken at 1‐min intervals and the average of these readings was used as the baseline resting measurement (Whelton et al. 2018).

#### 2.3.2 Ambulatory Blood Pressure Measurement

Ambulatory blood pressure was measured with fully automated BP monitors (ABPM‐06, Meditech, Hungary) programmed with CardioVisions software (Version 1.30.0, Meditech, Hungary). Devices were programmed to record BP at 30‐min intervals during daytime and 60‐min intervals at night‐time with both time periods individually defined by participant. During measurement, participants were advised to cease movement, remain still, and relax their hand by their side (Williams et al. 2018). If participants were seated, they were recommended to support their arm at heart level. Participants wore devices on nonworking days and were advised that for day of measurement they were not to participate in sport or exercise but to continue usual habitual behaviours. On return of the device, participants verbally confirmed sleep periods were unchanged.

#### 2.3.3 Blood Pressure Variability

To assess short‐term blood pressure variability (BPV), the average real variability (ARV) index was calculated for each individual 24‐h blood pressure measurement (Aizpu et al. 2005).

#### 2.3.4 Ambulatory Arterial Stiffness Index

To calculate the ambulatory arterial stiffness index (AASI), a linear regression was fitted to each participant's unedited 24‐h ambulatory blood pressure profile, with dBP regressed on sBP, with AASI defined as 1 minus the resulting regression slope (Dolan et al. 2006).

#### 2.3.5 Physical Activity Measurement

The international physical activity questionnaire short‐form (IPAQ‐SF) was completed by each participant pre and post the 4‐week training intervention to confirm habitual activity levels were maintained. This provided support that any observed changes in BP may be attributed to the isometric training intervention rather than a consequence of changes in weekly physical activity (Gribble et al. 2025). Data were analysed through a scoring spreadsheet providing an automatic calculation of IPAQ results and individual activity reports. MET‐min per week for each activity domain were recorded, and total weekly MET‐min per week. Weekly physical activity levels were classified as low (failing to meet required MET‐min per week for moderately active), moderate (achieving at least 600 MET‐min per week) and high achieving at least 3000 MET‐min per week (Kim et al. 2012).

### 2.4 Isometric Resistance Training

Both exercise groups were required to complete 4 weeks of supervised IRT programme. Exercise sessions were completed 3 days·a week for 4 weeks. Each exercise session was supervised by the lead researcher, and sessions were separated by at least 48 h.

#### 2.4.1 Isometric Handgrip

Isometric handgrip exercise was completed using a digital handgrip dynamometer (Zona Plus, Zona Health, USA). Participants completed a 3‐s maximal contraction to determine MVC, once established participants completed 4 x 2‐min contractions of alternating hands at 30% MVC, interspersed with a 1‐min rest period. During each 2‐min contraction participants received standardised verbal feedback, delivered at 30‐s intervals and were instructed to use visual and auditory cues provided from the digital display of the dynamometer to ensure the correct intensity was adhered to. On completion of a full training bout (4 x 2‐min), a session was deemed successful if participants were able to maintain the contraction intensity (30% MVC) for at least 80% of the prescribed training session to the extent that individual repetitions were held for ∼96 s at 30% MVC (Correia et al. 2020). Adherence was presented visually on the IHG device and recorded after each individual training, and data exported from the IHG device at the end of each training week. Handgrip strength was determined by calculating the mean weekly MVC value for each hand, with differences in week 1–4 analysed to determine changes in handgrip strength across the intervention.

#### 2.4.2 Isometric Training Band

Participants completed the ITB exercises in the following order: chest fly, seated lunge, seated pull and the bicep curl and tricep extension (Wright et al. 2025). A fixed order sequence of the individual exercises was prescribed to ensure a consistent and standardised protocol across the intervention. As it has been observed that specific ITB exercises may elicit a larger HR response to a single IHG contraction when performed in isolation (Wright et al. 2025), standardising the exercise order reduces order effects disproportionately affecting the cumulative exercise load. Moreover, Wright et al. (2025) demonstrated that when examining complete ITB bouts, in which exercise order was randomised, cardiovascular responses remained modest and safe regardless of exercise sequence. In the present protocol, the seated pull was positioned towards the end of the bout to avoid front‐loading the possibility of an elevated cardiovascular response. A complete ITB bout consisted of a 2‐min contraction for each of the 4 exercises separated by a 2‐min rest period. Contraction intensity was regulated following findings from Wright et al. (2025) shown to be equivalent to 30% MVC of IHG. This required participants to complete each exercise at a perceived localised exertion (CR‐10) that represents 4 at min^−1^, which progressed to an RPE of 5 by the end of min^−2^. Participants were shown the CR‐10 scale throughout each contraction to support adherence to the required intensity, alongside a verbal reminder of the required CR‐10 value delivered at fixed 30‐s intervals, ensuring consistency across both isometric training modes.

### 2.5 Data Analysis

Statistical analysis was completed using the Statistical Package of Social Sciences for Windows (SPSS, Version 28.0 IBM, Armonk, New York). Data were assessed for parametric assumptions (Field 2017) with normality of distribution analysed through Shapiro–Wilk tests. Where the distribution of data were non‐normal (Night‐time HR), data were logarithmically transformed (Log^10^). For nonparametric data (dBP^Night‐time^), a nonparametric Friedman's test was completed to analyse differences within group and a Kruskal–Wallis to assess differences between groups with Wilcoxon signed‐rank tests used for a post hoc assessment. All other normally distributed data were analysed using two‐way mixed model ANOVAs (time [pre and post] x group [CON, ITB and IHG]) to assess interaction effects and main effects for time and group. Moreover, absolute differences (post–pre) were analysed using a one‐way ANOVA with independent *t*‐tests used for the post hoc assessment. Sphericity assumption was satisfied as the repeated measure treatment variable consisted of two levels (pre and post). Estimates of effect sizes were calculated using partial eta squared (*η*_p_^2^) with Cohen's *d* calculated for all pairwise comparison and interpreted as small (0.2–0.49), medium (0.5–0.79) and large (> 0.8) (Cohen 1992). Moreover, For the CON group, all BP variables were analysed for group variation calculated by coefficient of variation (CoV) expressed as a percentage. Between group variable data, change data and results of pairwise comparisons were reported as mean ± standard error (SE). Alpha was set at 0.05 for all analysis.

## 3 Results

### 3.1 Participant Baseline Characteristics

There were no significant differences between groups at baseline for all measures (*p* > 0.05, Table 1). Self‐reported physical activity did not significantly change over the course of the intervention with no significant main effect of time, (*F*_(1, 39)_ = 0.011, *p* = 0.916), interaction effect (*F*_(2, 39)_ = 1.847, *p* = 0.171) or main effect of group (*F*_(2, 39)_ = 0.294, *p* = 0.747). Adherence for both exercise groups (ITB and IHG) was 100% with both successfully completing the twelve supervised training sessions over the 4‐week intervention. No change in MVC was observed between week‐1 and week‐4 for IHG (51.9 ± 2.1 vs. 51.2 ± 1.9, *p* > 0.05), with participants achieving an average of 93.2% from the 80% requirement for maintaining IHG contraction intensity. In both exercise groups, no adverse events were reported. The reliability of measurement for CON were (CoV with 95% CI) resting sBP 1.1% (0.4%–1.8%), dBP 4.8% (3.0%–6.6%), mBP 2.9% (1.6%–4.3%), HR 5.0% (3.2%–6.8%), sBP^24Hour^ 2.5% (0.7%–4.2%), sBP^Daytime^ 2.5% (0.8%–4.3%) and sBP^Night‐time^ 2.9% (1.1%–4.8%).

### 3.2 Resting Blood Pressure Measures

**FIGURE 2:**
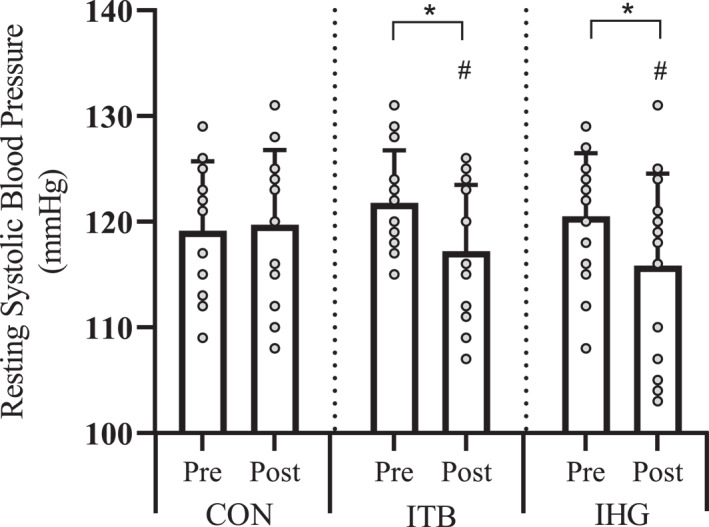
Changes in resting systolic blood pressure pre to postintervention in control (CON), isometric training band (ITB) and isometric handgrip (IHG) groups. Columns represent mean and SD with individual data points overlaid. * indicates a significant within‐group difference (*p* < 0.05). # indicates significant difference compared to CON (*p* < 0.05).

There was a significant interaction effect for resting sBP (*F*_(1, 39)_ = 13.35, *p* = < 0.001, *η*_p_^2^ = 0.406). Post hoc pairwise comparisons revealed significant reductions for IHG (−4.6 ± 3.6 mmHg, *p* < 0.05) and ITB (−4.5 ± 3 mmHg, *p* < 0.05) but not CON (0.57 ± 0.8 mmHg, *p* = 0.489). Group mean reductions in resting sBP were significantly greater than CON for both training groups ITB (−5.1 ± 1.2 mmHg, *p* = < 0.001, *d* = 1.89) and IHG (−5.2 ± 1.6 mmHg, *p* = < 0.001, *d* = 1.70). However, there were no significant differences in the magnitude of BP reduction between training groups (0.071 ± 1.1 mmHg, *p* = > 0.05, *d* = 0.021, Figure 2). For resting dBP, there were no main effect for time (*F*_(1,39)_ = 0.440, *p* = 0.511, *η*_p_^2^ = 0.011), no significant interaction effect (F _(2,39)_ = 700, *p* = 0.503, *η*_p_^2^ = 0.035) or main effect of group (*F*_(2,39)_ = 1.02, *p* = 0.368, *η*_p_^2^ = 0.050, Table 2). Group mean reductions in resting dBP were not significantly different between groups (*F*_(2,39)_ = 0.902, *p* = < 0.414, *η*_p_^2^ = 0.044).

**TABLE 2:** Within group pre and post, resting and ambulatory blood pressure changes.

	Control			Isometric training band			Isometric handgrip		
	Pre	Post	Δ	Pre	Post	Δ	Pre	Post	Δ
Resting									
dBP (mmHg)	73 ± 8	74 ± 6	1 ± 5	71 ± 7	70 ± 6	−1 ± 6	74 ± 7	72 ± 10	−2 ± 6
mBP (mmHg)	89 ± 7	89 ± 6	1 ± 4	88 ± 5	86 ± 5	−2 ± 4	90 ± 6	87 ± 6	−3 ± 5
HR (bpm^·−1^)	68 ± 11	67 ± 12	−1 ± 6	66 ± 12	66 ± 13	−0.4 ± 8	68 ± 14	67 ± 8	−1 ± 8
Ambulatory									
sBP^24Hour^	123 ± 10	125 ± 13	2 ± 5	123 ± 9	122 ± 9	−1.1 ± 5	127 ± 12	125 ± 12	−2 ± 7
dBP^24Hour^	73 ± 7	71 ± 4	2 ± 4	71 ± 4	72 ± 4	−1 ± 2	76 ± 6	76 ± 6	−0.2 ± 3
sBP^Daytime^	126 ± 10	128 ± 13	2 ± 6	126 ± 9	130 ± 11	−0.2 ± 5	130 ± 11	129 ± 12	−0.8 ± 7
dBP^Daytime^	75 ± 7	74 ± 4	2 ± 4	74 ± 3	78 ± 6	0.5 ± 3	78 ± 6	79 ± 6	0.3 ± 4
sBP^Night‐time^	113 ± 10	117 ± 15	4 ± 7	113 ± 9	109 ± 8	−4 ± 6^a^	118 ± 16	114 ± 13	−4 ± 7^a^
dBP^Night‐time^	64 ± 7	66 ± 8	4 ± 3	63 ± 6	61 ± 7	−1 ± 4	67 ± 6	65 ± 6	−2 ± 5

*Note:* Data are presented as mean ± SD.

Abbreviations: Δ, indicates magnitude of change pre‐to‐post; dBP, diastolic blood pressure; HR, heart rate; IHG, isometric handgrip; ITB, isometric training band; mBP, mean arterial pressure; sBP, systolic blood pressure.

^a^ Indicates a significant difference to pretraining *p* < 0.05.

### 3.3 Resting mBP

Equally, for resting mBP, there were no main effect for time (*F*_(1,39)_ = 3.29, *p* = 0.077, *η*_p_^2^ = 0.078) and no significant interaction effect between time and group (F _(2,39)_ = 2.023, *p* = 0.146, *η*_p_^2^ = 0.094), alongside no main effect of group (*F*_(2,39)_ = 0.543, *p* = 0.585, *η*_p_^2^ = 0.027) with the magnitude of change not significant between groups (F_(2,39)_ = 2.898, *p* = 0.067, *η*_p_^2^ = 0.129).

### 3.4 Resting HR

For resting HR, there were no interaction effects (*F*_(2,39)_ = 0.003, *p* = 0.997, *η*_p_^2^ = 0.000) or main effects of time (*F*_(1,39)_ = 0.088, *p* = 0.768, *η*_p_^2^ = 0.002) or group (*F*_(2,39)_ = 0.125, *p* = 0.883, *η*_p_^2^ = 0.006, Table 2) with the magnitude of change not significant between groups (F_(2,39)_ = 0.008, *p* = 0.993, *η*_p_^2^ = 0.01).

### 3.5 Ambulatory Blood Pressure Measures

Attrition for CON, ITB and IHG for AmBP measurement were 7.4% (13/14) 14.29% (12/14) and 7.4% (13/14), respectively. Causes of attrition were reported to be participant discomfort, failure to wear on a nonworking day and a failure to achieve the required number of measurements necessary for data analysis.

### 3.6 Ambulatory 24‐Hour Measurement

No interaction effects (*F*_(2,35)_ = 0.711–2.811, *p* = 0.074–0.498, *η*_p_^2^ = 0.039–0.138) or main effects of time (*F*_(1,35)_ = 0.011–1.894, *p* = 0.177–0.918, *η*_p_^2^ = 0.006–0.051) or group (*F*_(2,35)_ = 0.294–1.583, *p* = 0.220–0.747, *η*_p_^2^ = 0.017–0.083) were identified for 24‐h dBP, mBP and HR (Table 2).

### 3.7 Ambulatory Daytime Measurement

No interaction effects (*F*_(2,35)_ = 0.136–0.873, *p* = 0.414–873, *η*_p_^2^ = 0.008–0.049) or main effects of time (*F*_(1,35)_ = 0.084–2.531, *p* = 0.121–0.773, *η*_p_^2^ = 0.002–0.067) or group (*F*_(2,35)_ = 0.080–1.739, *p* = 0.191–923, *η*_p_^2^ = 0.005–0.090) were observed for daytime sBP, dBP, mBP or HR (Table 2).

### 3.8 Ambulatory Night‐Time Measurement

**FIGURE 3:**
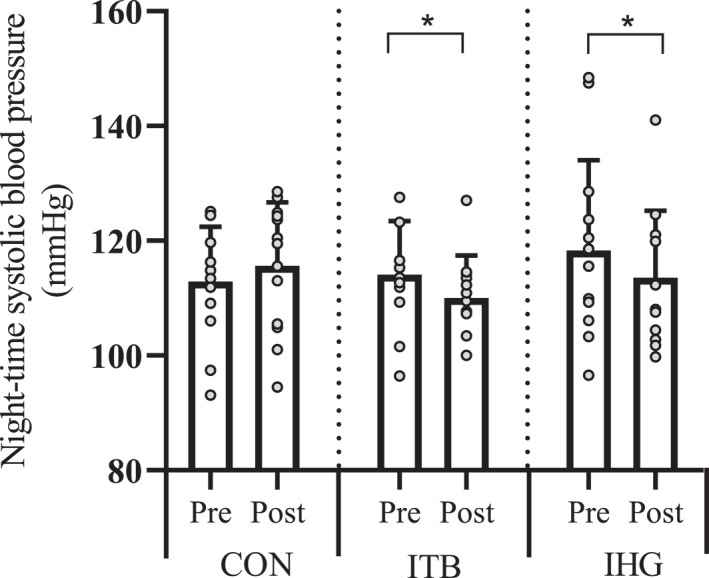
Changes in night‐time systolic blood pressure pre to postintervention in control (CON), isometric training band (ITB) and isometric handgrip (IHG) groups. Columns represent mean ± SD with individual data points overlaid. * indicates a significant within‐group difference (*p* < 0.05).

A significant interaction effect was revealed for sBP^Night‐time^ (*F*_(2,35)_ = 5.256, *p* = 0.010, *η*_p_^2^ = 0.231). Post hoc pairwise comparisons revealed significant reductions (*p* < 0.05) in both training groups (ITB −4 ± 1.8 mmHg, IHG, −4.7 ± 1.8 mmHg). Group mean reductions were significantly greater than CON (*F*_(2,35)_ = 5.347, *p* = < 0.009, *η*_p_^2^ = 0.234), for the ITB (−6.9 ± 2.6 mmHg, *p* = 0.036, *d* = 1.17) and IHG (−7.6 ± 2.5 mmHg, *p* = 0.016, *d* = 1.2), though between training groups there was no significant difference (0.67 ± 2.6 mmHg, *p* = > 0.05, *d* = 0.094. Figure 3).

There were no significant differences in dBP^Night‐time^ between groups at baseline χ^2^(2) = 4.052, *p* = 0.132 or post‐4 weeks of IRT χ^2^(2) = 4.519, *p* = 0.104. With no significant differences across time within groups χ^2^(1) = 0.243 and *p* = 0.622. Similarly, no interaction effects (*F*_(2,35)_ = 1.023, *p* = 0.370, *η*_p_^2^ = 0.055) or main effects of time (*F*_(1,35)_ = 0.113, *p* = 0.738, ηp^2^ = 0.003) or group (*F*_(2,35)_ = 0.137, *p* = 0.872, *η*_p_^2^ = 0.008) were identified for night‐time HR(^Log10^).

### 3.9 Ambulatory Arterial Stiffness Index

There was a significant interaction effect between time and group (*F*_(2,35)_ = 3.359, *p* = 0.046, *η*_p_^2^ = 0.161) with no main effect of time (*F*_(1,35)_ = 0.028, *p* = 0.896, *η*_p_^2^ = 0.001) or main effect of group (*F*_(2,35)_ = 1.459, *p* = 0.246, *η*_p_^2^ = 0.077). Pairwise comparisons identified a significant increase in AASI for the control group (0.36 ± 0.04 vs. 0.48 ± 0.05, *p* = 0.043, *d* = 0.57), with no significant pre‐to‐post changes observed in the training groups. Analysis of change scores showed no significant differences between groups (F_(2,37)_ = 0.253, *p* = 0.094, *η*_p_^2^ = 0.126).

### 3.10 Blood Pressure Variability

For systolic ARV, there were no interaction effects (*F*_(2,35)_ = 0.505, *p* = 0.608, *η*_p_^2^ = 0.028) alongside no main effects for time (*F*_(1,35)_ = 0.046, *p* = 0.831, *η*_p_^2^ = 0.001) or group (*F*_(2,35)_ = 0.038, *p* = 0.963, *η*_p_^2^ = 0.002) alongside no significant differences in absolute change scores (F_(2,37)_ = 0.328, *p* = 0.722, *η*_p_^2^ = 0.018). Similarly, for diastolic ARV, no interaction effects were observed (*F*_(2,35)_ = 0.761, *p* = 0.475, *η*_p_^2^ = 0.042) with no main effect for time (*F*_(1,35)_ = 1.064, *p* = 0.309, *η*_p_^2^ = 0.029) or group (*F*_(2,35)_ = 1.212, *p* = 0.310, *η*_p_^2^ = 0.065) alongside no significant between‐group absolute differences (F_(2,37)_ = 0.761, *p* = 0.475, *η*_p_^2^ = 0.042).

## 4 Discussion

The primary aims of this study were to establish whether a 4‐week novel ITB exercise intervention reduces resting and AmBP and to examine BP changes following contraction volume‐matched IRT, specifically comparing the ITB with a validated IHG protocol. Findings suggest 4 weeks of IRT performed with the ITB can induce significant reductions in resting sBP and night‐time sBP in a normotensive cohort that are comparable to an established IHG protocol. Moreover, to the authors' knowledge, this is the first study to report significant reductions in night‐time sBP following 4 weeks of IRT in a normotensive cohort. These findings provide support for the ITB being an effective, practical and cost‐effective alternative IRT mode for reducing resting and night‐time sBP.

### 4.1 Reductions in Resting Measures

The significant reductions in resting sBP for both exercise groups are in agreement with those reported in meta‐analyses (Carlson et al. 2014; Inder et al. 2016) and studies implementing an equivalent IHG protocol (Badrov et al. 2013). Moreover, comparable reductions in sBP are reported in 4‐week IRT studies utilising larger muscle groups and prescribing 96 min of total isometric contraction volume (Devereux et al. 2010; Wiles et al. 2017). Collectively, these findings strengthen the evidence that 4 weeks of IRT totalling 96 min of contraction volume can elicit significant reductions in resting sBP independent of isometric mode, further supporting IRT as a time‐efficient exercise modality in hypertension management. Findings evidence efficacy for the ITB protocol as a novel therapeutic IRT intervention with equivalent sBP reductions to established IHG methods. However, significant reductions in dBP and mBP were not observed for either exercise group (Table 2), though not an uncommon finding in IRT research (Baross et al. 2012; Carlson et al. 2016; Gordon et al. 2018; Hess et al. 2016) and may be attributed to baseline values being in normal ranges for both exercise groups (ITB, 71 mmHg and IHG, 74 mmHg) thusly limiting capacity for reduction (Carlson et al. 2014).

### 4.2 The Impact of Exercising Muscle Mass

Given muscle mass is suggested to be a variable influencing IRT‐induced BP reductions (Lawrence et al. 2015), surprisingly the ITB did not elicit superior sBP decreases compared to the IHG. However, this may be explained by the ITB protocol being purposefully designed to elicit a haemodynamic response equivalent to IHG (Wright et al. 2025). Larger reductions in sBP have been observed in studies prescribing IRT of larger muscle mass (Decaux et al. 2022; Lea et al. 2023), though in a prehypertensive population (resting sBP 131 & 134 mmHg, respectively). Variation in the intervention should be acknowledged as the aforementioned studies prescribed repeat contractions of the same muscle groups, producing a consistent cardiovascular stress to the working muscle and local vasculature. In contrast, due to the multiexercise approach of the ITB, each individual bout would have elicited a diminished localised blood flow, shear and haemodynamic stress aligned with the reduced contraction volume for each individual exercise. Over the course of the 4‐week intervention, each individual ITB exercise was prescribed as 24‐min of contraction volume as opposed to 96‐min for singular‐exercise modes. This training volume disparity could have implications on vasculature adaptations through repeat exposure to haemodynamic shear stress. Accordingly, it is of interest to examine if longer application of the ITB protocol may result in typical time‐dependent phasic vascular adaptations (Tinken et al. 2010) Furthermore, as within‐session cardiovascular responses were not recorded, the subsequent load may have been variable between participants, which may relate to the interindividual variability in BP reductions observed with IRT (Kelley et al. 2021). Also, as the ITB protocol has only safely been established in normotensives (Wright et al. 2025), prior to application in pre and hypertensive populations, assessment of the acute response is necessary to prevent any heightened risk of CV events especially as baseline BP may impact the magnitude of the pressor response (Greaney et al. 2014).

### 4.3 Changes in Ambulatory Blood Pressure Measures

It is suggested that changes in night‐time sBP provide greater prognostic value for cardiovascular events and all‐cause mortality than daytime or resting BP (Staplin et al. 2023). The significant reductions in night‐time sBP combined with the cost of the ITB are encouraging, and further provide evidence for the efficacy of this novel method for IRT. The magnitude of reductions for night‐time sBP agree with those reported following traditional exercise modes (Saco‐ledo et al. 2020) and are comparable to other novel IRT modalities (Lea et al. 2023). Notably, the ITB protocol employed in the current study adopted a similar method of intensity prescription to that used by Lea et al. (2021) by utilising RPE, further supporting its potential as an effective and accessible alternative method for IRT intensity regulation. The absence of changes in 24‐h and daytime AmBP indices is not uncommon in IRT studies (Goessler et al. 2018; Pagonas et al. 2017; Stiller‐Moldovan et al. 2012) and may be attributed to various factors, including, dropout and attrition rates, absence of a full habituation period (Kleiber et al. 2023) alongside baseline sample characteristics. In the present study, attrition in the ITB group was double that of the IHG group, which may have contributed to the observed AmBP results. Some participants were unable to complete AmBP measurements due to discomfort though not an uncommon issue when faced with repeated cuff inflation (Thomsen et al. 2023). Furthermore, daily recordings may have been influenced by cuff‐inflation induced hypertension with repeated measurements elevating average daily pressure (Charmoy et al. 2007). Reduced measurement frequency is seen to reduce cuff‐related discomfort (Thomsen et al. 2023) with low‐frequency (every 60‐min) showing better association with resting BP (Dabrowski et al. 2021). Yet, the current study adopted an established protocol (Williams et al. 2018) with the frequency of recordings allowing measurement of other parameters such as AASI and BPV. This change of protocol may require the adoption of a 48‐h measurement period, nevertheless longer recording periods are uncommon in clinical practice and participant comfort, adherence and measurement attrition need to be considered.

### 4.4 Effects of Isometric Resistance Training on Blood Pressure Variability and Ambulatory Arterial Stiffness Index

Irrespective of exercise mode, the present study found 4‐week of IRT did not alter BPV which contrast the limited existing literature and likely the result of contrasting methodological approaches as both Baross et al. (2022) and Taylor et al. (2019) prescribed a longer (8‐week) intervention with the latter sampling unmedicated hypertensives. Whereas, the current study sampled normotensives participants who may have limited capacity for change in factors influencing short‐term BPV (Chadachan et al. 2018) such as baroreceptor reflex sensitivity, vascular dysfunction and sympathetic activity (Parati et al. 2020). On the contrary, longer interventions (≥ 8 weeks) have shown significant changes parallel with improved baroreceptor reflex sensitivity and markers of vascular function (Taylor et al. 2019) that agree with time‐course patterns of vascular adaptation (Tinken et al. 2010).

This is the first study to examine changes to AASI following an exercise intervention as earlier studies have focused on acute exercise responses (Michalski et al. 2023). The absence of AASI changes in both exercise groups suggests a lack of peripheral vascular adaptation, likely due to the intervention's short duration being insufficient to trigger the biphasic pattern of vascular remodelling. Additionally, baseline AASI values were within the normal range < 0.50 (Li et al. 2006), potentially limiting the scope for adaptation. Interestingly, the CON group displayed a significant increase without a corresponding increase in resting BP or AmBP, which is unexpected given the contribution of peripheral resistance to BP. Although unexpected, this may be a consequence of the increased 24‐h dBP postintervention as the AASI is derived from the regression of dBP on sBP, its value is sensitive to the variability of the 24‐h profile of dBP (Schillaci and Pucci 2015). Moreover, authors cannot rule out this finding being subjective to variability in reproducibility of the AASI (Kollias et al. 2012). Kollias et al. (2015) found no changes in AASI despite significant reductions in AmBP and pulse wave velocity following antihypertensive treatment, suggesting AASI may be affected by alternative mechanisms (Schillaci and Pucci 2015) which warrants consideration if used interchangeably as a measure of arterial stiffness and compliance. Though without a direct assessment of arterial stiffness caution is required when associating these findings to theoretical BP‐reducing mechanisms.

### 4.5 Limitations and Future Directions

A limitation of the present study is that findings are limited to a healthy normotensive population, thusly not representative of pre and hypertensive adults. Moreover, authors cannot rule out changes to physical activity during measurement and other confounding psychological, and environmental factors potentially influencing AmBP readings (Brook et al. 2011; Tomitani et al. 2023). This may be a possible reason for the disparity in BP reductions between resting, night‐time and 24‐h and daytime AmBP. Laboratory clinical‐based measurement allows a greater control of external factors allowing mitigation of environmental stressors known to cause transient increases and fluctuations of BP (Brook et al. 2011).

Given the multiexercise capability of the ITB future interventions may prescribe individual exercises to optimise the precision of exercise and enhance enjoyment, aligning with the growing interest on personalised exercise prescription, recently emphasised by the European Society of Cardiology (Hanssen et al. 2021). However, further research is warranted to establish the BP‐lowering ability of additional ITB exercises of varied prescription. Findings are promising as observed reductions are found in normotensives who typically exhibit a limited capacity for BP change compared to pre and hypertensive populations (MacDonald and Pescatello 2018) and in active adults to whom the prevalence of hypertension is rising (Hamrahian and Falkner 2022). Moreover, all exercises performed with the ITB were completed in a seated position increasing the potential application for IRT in those with limited mobility.

## 5 Conclusion

Overall, these findings suggest that a 4‐week ITB protocol can elicit reductions in resting and night‐time sBP in normotensives comparable to IHG and provide additional evidence supporting the efficacy of IRT as a BP‐lowering exercise modality. The comparable BP reductions between training methods coupled with absence of any adverse events are encouraging for the ITB as a novel approach to IRT in normotensive adults. As such, the ITB may offer a practical and cost‐effective alternative to long‐established IRT methods.

## Author Contributions

All authors contributed to the study conception and design. Material preparation, data collection and analysis were performed by the lead author. The first draft of the manuscript was written by the lead author and all authors commented on previous versions of the manuscript. All authors have read and approved the final manuscript for publication.

## Funding

The authors have nothing to report.

## Ethics Statement

Institutional ethical approval was obtained (ETH1920‐ 0152) from the University of Northampton Research Ethics Panel in accordance with the latest Declaration of Helsinki.

## Conflicts of Interest

The authors declare no conflicts of interest.

## Data Availability

The datasets generated during and analysed from the current study are available from the corresponding author on reasonable request.
